# Double Trouble: Ductal Origin of Right Pulmonary Artery With Contralateral Embolism

**DOI:** 10.1016/j.cjcpc.2022.02.002

**Published:** 2022-02-11

**Authors:** Alessandro Pierri, Antonio De Luca, Luca Restivo, Alessandro Bologna, Davide Barbisan, Manuel Belgrano, Francesca Marchetti, Gianluca Pontone, Gianfranco Sinagra

**Affiliations:** a Cardiothoracovascular Department, Azienda Sanitaria Universitaria Giuliano Isontina, University of Trieste, Trieste, Italy; b Department of Radiology, Azienda Sanitaria Universitaria Giuliano Isontina, University of Trieste, Trieste, Italy; c Cardiovascular Imaging Department, Centro Cardiologico Monzino IRCCS, University of Milan, Milan, Italy

## Case

**Figure 1:**
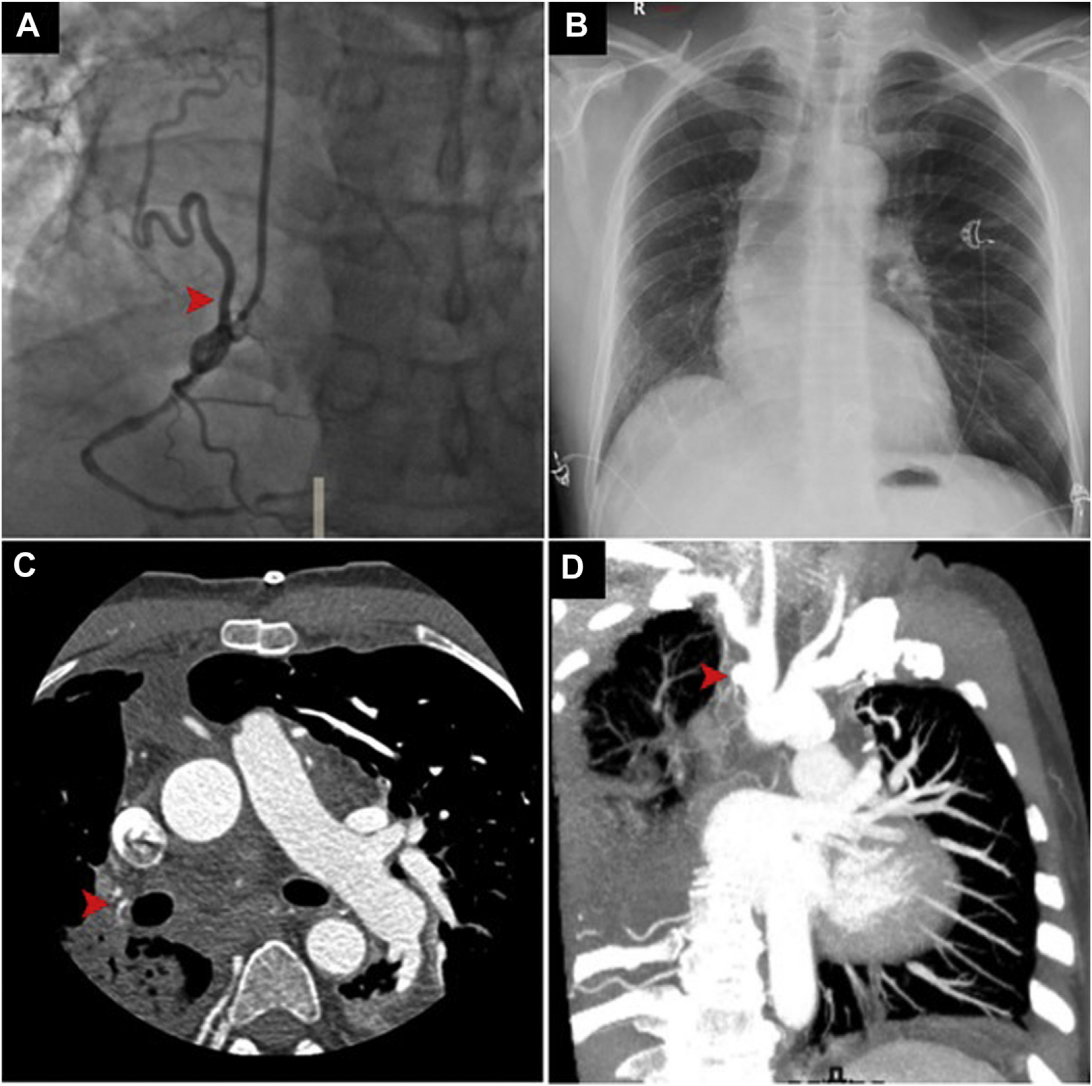
(**A**) Right coronary angiogram revealing an anomalous collateral originating from the right coronary artery (**arrowhead**). (**B**) Chest radiograph showing asymmetric lung volumes, mediastinal shift towards the right and ipsilateral elevated hemidiaphragm. (**C**) Axial angiogram demonstrating proximal interruption of the right pulmonary artery while the distal pulmonary arterial tree is maintained (**arrowhead**). (**D**) Coronal angiogram showing the telltale sign of ductal origin of pulmonary artery, namely, the ductal diverticulum (**arrowhead**).

**Figure 2:**
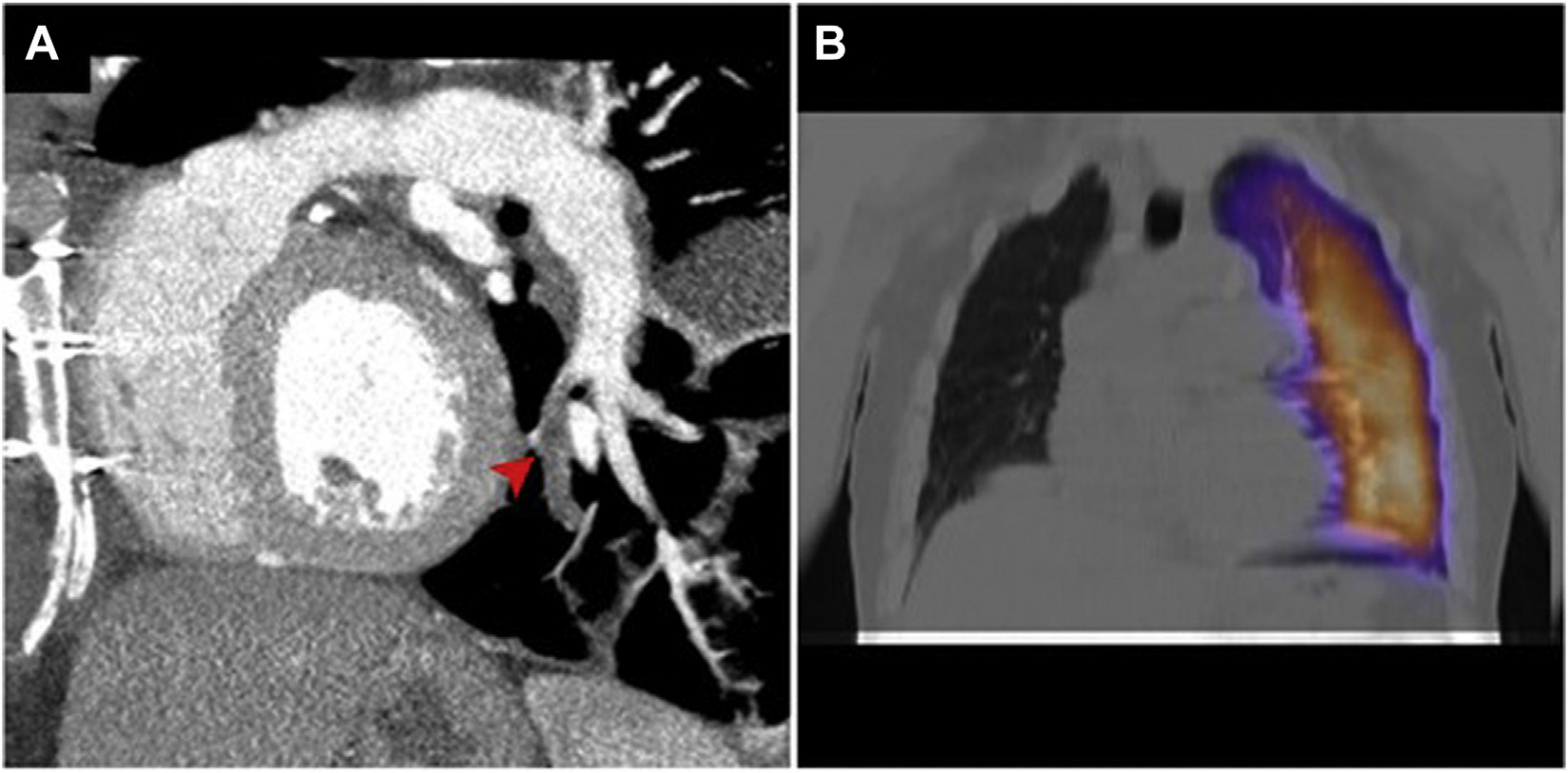
(**A**) Sagittal angiogram revealing a pulmonary embolus in the posteromedial basal segmental branch of the left pulmonary artery (**arrowhead**). (**B**) Lung perfusion scan showing no radioisotope activity in the right lung and normal perfusion of the left lung.

A 60-year-old male patient underwent coronary angiography for acute myocardial infarction. Angiograms revealed significant multivessel disease and an abnormal branch arising from the proximal right coronary artery and extending backwards, likely to the right lung (Fig. 1A). Both internal thoracic arteries were patent; notably, the calibre of the right one was markedly larger than the left. Although chest radiograph showed anomalies such as right-lung hypoplasia and ipsilateral mediastinal shift (Fig. 1B), further investigation was skipped as the patient underwent urgent coronary artery bypass grafting. The postoperative course was complicated by respiratory impairment; thus a computed tomography pulmonary angiogram (CTPA) was performed, revealing the absence of the mediastinal segment of the right pulmonary artery (PA) (Fig. 1C). Interestingly, a small vascular outpouching representing the remnant lumen of a closed right-sided ductus arteriosus was identified at the base of the right innominate artery (Fig. 1D). Based on these findings, the patient was diagnosed with isolated ductal origin of pulmonary artery (DOPA). A left lower lobe segmental pulmonary embolism (PE) was also identified as the precipitating cause of respiratory distress (Fig. 2A); therefore, heparin was started. Two weeks later, a lung perfusion scan showed the resolution of PE (Fig. 2B).

## Discussion

Isolated DOPA is a rare vascular malformation that may remain undiagnosed until advanced age, often manifesting with nonspecific symptoms such as recurrent bronchopneumonia, haemoptysis, and dyspnea.^1^ In the present case, the ventilation-perfusion mismatch associated with DOPA was exacerbated by contralateral PE, potentially explaining the postoperative respiratory distress.

Lack of a connection between the main and affected branch PA, resulting from the involution of the ventral sixth aortic arch, led to the erroneous definition of unilateral pulmonary artery agenesis.^1^^,^^2^ Instead, the term DOPA more accurately describes this malformation, as a well-formed, albeit hypoplastic, true pulmonary artery is invariably present, the hilar PA being connected to the dorsal sixth aortic arch (ductus arteriosus) in foetal life.^1^ After birth, the ductus usually involutes to become the ligamentum arteriosum, sometimes appearing on angiograms as a small outpouching from the innominate or subclavian artery ipsilateral to the affected side. Subsequently, the affected lung can be supplied by compensatory systemic collaterals originating from bronchial, internal thoracic, and coronary arteries,^2^ as our case shows.

Interestingly, DOPA could be suspected by jointly considering the anomalies detected by coronary angiography and chest radiograph. Despite not providing direct visualization of DOPA, these diagnostic modalities could offer subtle but important clues to this rare malformation, ultimately confirmed by CTPA.

**Novel Teaching Points**

• Chest radiograph may provide relevant clues to the diagnosis of DOPA.
• CTPA is the method of choice for noninvasive diagnostic confirmation.
• Key diagnostic findings are a well-formed but hypoplastic pulmonary artery and a ductal diverticulum on the aortic arch or the base of an epiaortic vessel.
